# MIxS-BE: a MIxS extension defining a minimum information standard for sequence data from the built environment

**DOI:** 10.1038/ismej.2013.176

**Published:** 2013-10-24

**Authors:** Elizabeth M Glass, Yekaterina Dribinsky, Pelin Yilmaz, Hal Levin, Robert Van Pelt, Doug Wendel, Andreas Wilke, Jonathan A Eisen, Sue Huse, Anna Shipanova, Mitch Sogin, Jason Stajich, Rob Knight, Folker Meyer, Lynn M Schriml

**Affiliations:** 1 Mathematics and Computer Science Division, Argonne National Laboratory, Argonne, IL, USA; 2 Max Planck Institute for Marine Microbiology, Bremen, Germany; 3 Building Ecology; 4 Department of Computer Science, University of Colorado, Boulder, CO, USA; 5 Davis Genome Center, University of California, Davis, CA, USA; 6 Marine Biological Laboratory, Woods Hole, MA, USA; 7 University of California, Riverside, CA, USA; 8 Howard Hughes Medical Institute, Chevy Chase, MD, USA; 9 Institute for Genomics and Systems Biology, Argonne National Laboratory, Argonne, IL, USA; 10 Department of Epidemiology and Public Health, Institute for Genome Sciences, University of Maryland School of Medicine, Baltimore, MD, USA

## The need for metadata standards for microbe sampling in the built environment

The composition of indoor microbial communities has the potential to profoundly affect human health. A number of factors within a building or room can alter the microbial abundance and diversity, such as occupancy, temperature and humidity, which in turn impacts indoor air quality. Researchers Hospodsky *et al.* (2012); Kembel *et al.* (2012) and Dunn *et al.* (2013) are exploring the intersection of microbial ecology, building materials and architectural design to understand microbial diversity and abundance within a building. Metadata (data describing data) provides an essential complement to experimental data, helping to answer questions about its source, mode of collection and reliability. As the impact and prevalence of large-scale metagenomic surveys grow, so does the need for more complete and standards compliant metadata (Gilbert *et al.*, 2012). While marine, soil and the human microbiome environments have representation in the standards being developed the built environment (BE) represents a unique context in need of standards development for the use in the study of microbial sequences. Metadata collection and interpretation have become vital to the genomics and metagenomics community to share information and integrate data across resources and within data repositories. The Genomic Standards Consortium (GSC, http://gensc.org; Field *et al.* (2011)) has developed widely accepted metadata MIxS (MIGS, MIMS, MIMARKS; Yilmaz *et al.* (2011)) standards for genomic, metagenomic and amplicon (for example, 16S rRNA) sequence data sets. These standards have been developed within a framework that is both modular and extensible. The MIxS-BE, as a minimal metadata standard, represents a unique extension to the GSC's MIxS standard as a rigorous and structured tool for the analysis of microbial sequences and ecosystems of the indoor environment. The MIxS-BE package provides the BE community with a suggested list of parameters to record and report for each sequenced sample and to compare data across studies. The MIxS-BE standard incorporates the core set of required MIxS fields for a bacterial sequence along with a Built Environment (BE) package (http://gensc.org/index.php?title=MIxS_extensions) comprised of a BE core, MIxS air environmental package terms, BE building properties and BE sample properties. The MIxS-BE package has been incorporated into the MIxS checklist and integrated into the QIIME (Caporaso *et al.*, 2010), MoBeDAC and MG-RAST (Meyer *et al.*, 2008) databases to foster metadata submission compliance across BE projects. Updates to the MIxS specification will be included in BioSample (Barrett *et al.*, 2012) as part of the annual MIxS release. Requests for additions or changes to the MIxS checklists can be directed to the GSC Developers mailing list: "mailto:developers@gensc.org or by contacting Lynn Schriml or Elizabeth Glass.

Development of the MIxS-BE package has been an open and iterative process engaging the GSC community, the GSC's MIxS developers, stakeholders across the BE community including microbial ecologists, microbiologists, architects and engineers. The Microbiology of the Built Environment Alfred P. Sloan funded initiative established a metadata working group as part of the MoBeDAC, bringing together Sloan-funded researchers, architects, civil engineers, bioinformaticists and computational biologists to discuss the need and context for standards to describe the most relevant metadata to be collected for BE samples. Led by Elizabeth Glass and Lynn Schriml, the working group proposed the development of the BE-MIxS package to the GSC and gained the GSC's board approval to develop the standard. The working group initially identified a comprehensive list of metadata terms reported in BE literature. Based on feedback solicited from industry experts and microbial ecologists, the working group established a minimal set of metadata terms for the BE package environment samples with a subset of the MIxS-BE terms classified as M (Mandatory), which indicates that the term has to be reported for the metadata to be considered compliant to the MIxS checklist. The MIxS-BE minimal set will be complemented by a MIxS-BE-Building package (under development) describing the larger set of building and room metadata pertinent to describing a BE sample (Table 1).

## Figures and Tables

**Table 1:** MIxS-BE metadata package terms

**MIxS-BE term**
Carbon dioxide^a^
Ventilation type^a^
Organism count^a^

BE core
Surface material
Surface-air contaminant
Relative air humidity
Absolute air humidity
Surface humidity
Air temperature
Surface temperature
Surface moisture pH
Surface moisture
Dew point
Building occupancy type

BE Building properties
Indoor space (room type)
Indoor surface
Filter type
Heating and cooling system type
Substructure type
Building setting
Light type

BE Sample properties
Sample size sorting method
Space typical state
Typical occupant density
Occupancy at sampling
Occupant density at sampling

Terms, definitions, required or optional status (for reporting compliance) and syntax are presented at: MIxS project, (http://gensc.org/gc_wiki/images/7/70/Built_environment-metadata-terms-v51.xls).

a The MIxS-BE package includes MIxS-air environmental package terms and the built environment core, building and sample property terms.

## References

[bib1] Barrett T Clark K Gevorgyan R Gorelenkov V Gribov E Karsch-Mizrachi I 2012 BioProject and BioSample databases at NCBI: facilitating capture and organization of metadata Nucleic Acids Res 40Database issueD57 D63 22139929 10.1093/nar/gkr1163 PMC3245069

[bib2] Caporaso JG Kuczynski J Stombaugh J Bittinger K Bushman FD Costello EK 2010 QIIME allows analysis of high-throughput community sequencing data Nat Methods 7 335 336 20383131 10.1038/nmeth.f.303 PMC3156573

[bib3] Dunn RR Fierer N Henley JB Leff JW Menninger HL 2013 Home life: factors structuring the bacterial diversity found within and between homes PLoS One 8 e64133 23717552 10.1371/journal.pone.0064133 PMC3661444

[bib4] Field D Amaral-Zettler L Cochrane G Cole JR Dawyndt P Garrity GM 2011 The Genomic Standards Consortium PLoS Biol 9 e1001088 21713030 10.1371/journal.pbio.1001088 PMC3119656

[bib5] Gilbert JA Steele JA Caporaso JG Steinbrück L Reeder J Temperton B 2012 Defining seasonal marine microbial community dynamics ISME J 6 298 308 21850055 10.1038/ismej.2011.107 PMC3260500

[bib6] Hospodsky D Qian J Nazaroff WW Yamamoto N Bibby K Rismani-Yazdi H 2012 Human occupancy as a source of indoor airborne bacteria PLoS One 7 e34867 22529946 10.1371/journal.pone.0034867 PMC3329548

[bib7] Kembel SW Jones E Kline J Northcutt D Stenson J Womack AM 2012 Architectural design influences the diversity and structure of the built environment microbiome ISME J 6 1469 1479 22278670 10.1038/ismej.2011.211 PMC3400407

[bib8] Meyer F Paarmann D D'Souza M Olson R Glass EM Kubal M 2008 The metagenomics RAST server—a public resource for the automatic phylogenetic and functional analysis of metagenomes BMC Bioinformatics 9 386 18803844 10.1186/1471-2105-9-386 PMC2563014

[bib9] Yilmaz P Kottmann R Field D Knight R Cole JR Amaral-Zettler L 2011 Minimum information about a marker gene sequence (MIMARKS) and minimum information about any (x) sequence (MIxS) specifications Nat Biotechnol 29 415 420 21552244 10.1038/nbt.1823 PMC3367316

